# Hyperglycemia facilitates EV71 replication: Insights into miR-206-mediated regulation of G3BP2 promoting EV71 IRES activity

**DOI:** 10.7150/thno.93883

**Published:** 2024-04-22

**Authors:** Rai-Hua Lai, Yen-Hung Chow, Yi-Wen Lin, Nai-Hsiang Chung, Shu-Wei Nien, Jyh-Lyh Juang

**Affiliations:** 1 National Center for Geriatrics and Welfare Research, National Health Research Institutes, Miaoli, Taiwan.; 2 Institute of Molecular and Genomic Medicine, National Health Research Institutes, Miaoli, Taiwan.; 3 Institute of Infectious Disease and Vaccinology, National Health Research Institutes, Zhunan Town, Miaoli County, Taiwan.; 4 Graduate Institute of Biomedical Sciences, China Medical University, Taichung, Taiwan.; 5 Microarray Core Laboratory, National Health Research Institutes, Miaoli, Taiwan.

**Keywords:** hyperglycemia, neurovirulence, enterovirus, miR-206, internal ribosome entry site (IRES)

## Abstract

**Background:** Neurotropic virus infections actively manipulate host cell metabolism to enhance virus neurovirulence. Although hyperglycemia is common during severe infections, its specific role remains unclear. This study investigates the impact of hyperglycemia on the neurovirulence of enterovirus 71 (EV71), a neurovirulent virus relying on internal ribosome entry site (IRES)-mediated translation for replication.

**Methods:** Utilizing hSCARB2-transgenic mice, we explore the effects of hyperglycemia in EV71 infection and elucidate the underlying mechanisms.

**Results:** Remarkably, administering insulin alone to reduce hyperglycemia in hSCARB2-transgenic mice results in a decrease in brainstem encephalitis and viral load. Conversely, induced hyperglycemia exacerbates neuropathogenesis, highlighting the pivotal role of hyperglycemia in neurovirulence. Notably, miR-206 emerges as a crucial mediator induced by viral infection, with its expression further heightened by hyperglycemia and concurrently repressed by insulin. The use of antagomiR-206 effectively mitigates EV71-induced brainstem encephalitis and reduces viral load. Mechanistically, miR-206 facilitates IRES-driven virus replication by repressing the stress granule protein G3BP2.

**Conclusions:** Novel therapeutic approaches against severe EV71 infections involve managing hyperglycemia and targeting the miR-206-stress granule pathway to modulate virus IRES activity.

## Introduction

The rise of neurovirulent enteroviruses has evolved into a global public health concern. Infection among young children with potent neurotoxic strains like poliovirus and EV71 can lead to encephalitis, resulting in substantial morbidity and mortality 1, 2. Unfortunately, there is no effective antiviral treatment for EV71. Additionally, developing vaccines against these neurovirulent enteroviruses is challenging due to their single-stranded RNA nature, which allows for rapid mutations that can render vaccines ineffective 3. EV71 is a particularly neurovirulent serotype of enterovirus, known for its high efficiency in infiltrating and replicating within the central nervous system (CNS). Thus, a comprehensive understanding of the neurovirulence factors contributing to CNS damage is imperative for advancing therapeutic interventions.

Once EV71 infiltrates the CNS, it promptly undergoes replication, culminating in encephalitis characterized by an exaggerated immune reaction and cytokine storm 4. This cascade inflicts severe neurological complications and damage. Similar to other RNA viruses, enteroviruses are reliant on the host cell's machinery to produce viral proteins for replication and dissemination. Among neurotropic viruses, including enteroviruses, the IRES serves as a mechanism for initiating virus protein synthesis independently of the host cell's cap-dependent translation apparatus 5. Consequently, comprehending the factors influencing efficient IRES-dependent viral replication in the CNS is pivotal for the advancement of targeted therapies aimed at managing and mitigating their neurovirulent effects.

Host cell metabolism plays a crucial role in regulating virus neurovirulence. Given the energy-intensive processes involved in viral genome replication and synthesis of viral proteins, particularly in an environment typically unfavorable to the virus, the modulation of host cell metabolism becomes especially critical to sustain an infection in the CNS. Several host factors are likely contributors to the neurovirulence of EV71, encompassing cellular receptors, immune response, and genetic factors 6, 7. Intriguingly, hyperglycemia frequently emerges as a metabolic anomaly in encephalitis pathogenesis, and it also acts as a significant clinical indicator of brainstem encephalitis in severe EV71-infected children 8, 9. Hyperglycemia is associated with glycemic fluctuations, which are commonly observed during acute inflammatory responses in severe medical conditions 10. Consequently, intensive insulin therapy has become a standard clinical practice for patients in Intensive Care Units 11. Nevertheless, research in this domain is scant to conclusively support the proposition that hyperglycemia-triggered signaling pathways are pivotal in severe EV71 infection development.

Poliovirus, a prominent subtype of enterovirus, has been extensively studied as a neurotropic virus inducing CNS manifestations in young children 12. Deeper insight into poliovirus's neurovirulence mechanism can provide valuable perspectives for devising effective strategies against viral infections. Research on poliovirus neuropathology has revealed its close association with the IRES located within the viral genomic RNA's 5' untranslated region (UTR) 13. The viral-induced translational regulation, marked by the cleavage of eIF4G by poliovirus, hampers cap-dependent 5' UTR translation initiation in most eukaryotic mRNAs, consequently facilitating robust viral replication 14. Intriguingly, EV71 also features an IRES element that empowers EV71 translation to overtake cellular translation 15. However, the cellular mechanisms orchestrating the activation of viral IRES activity during EV71 infectious disease pathogenesis largely remain enigmatic.

Epidemiological studies suggest an association between enterovirus infection and type 1 diabetes 16, suggesting that enteroviruses may exhibit tropism for pancreatic islet cells, contributing to dysregulated glucose levels. However, it remains somewhat uncertain whether enterovirus infection-induced hyperglycemia influences virus neurotropism. The study was focused to explore the potential link between hyperglycemia and neurovirulence of EV71 using a mouse model. Our discoveries unveil that hyperglycemia amplifies EV71's neurovirulence through a miRNA-mediated signaling route, consequently prompting the activation of IRES-dependent viral protein synthesis. This insight could help in ongoing efforts to find different approaches for easing EV71-induced encephalitis, without solely relying on direct antiviral agents.

## Results

### EV71 Infection Causes Hyperglycemia in hSCARB2-Tg Mice

EV71 infection has been linked to acute pancreatitis in humans, contributing to hyperglycemia 17. To ascertain if a mouse model would also manifest a similar disturbance in blood glucose homeostasis upon EV71 infection, we administered a lethal dose of EV71 via intraperitoneal (i.p.) injection to hSCARB2-Tg mice, a transgenic model employed for studying enterovirus infections 18, 19. Indeed, EV71 infection in these mice yielded a notable elevation in blood glucose levels and a concurrent decline in plasma insulin levels, paralleling observations in human patients (Figure S1A-B). We hypothesized that EV71 infection targets and damages islet cells, leading to reduced insulin secretion. In support of this hypothesis, VP1/2 antibody staining revealed a concentration of EV71 particles within the islet tissues at 3 days post-infection (dpi) (Figure S1C). Histopathological scrutiny further unveiled lesions in the pancreatic tissues subsequent to EV71 infection (Figure S1D), indicative of potential islet cell damage due to the viral infection.

Given that compromised pancreatic islet cells could result in impaired insulin production and secretion, we probed whether the levels of glucokinase (GCK), a glucose sensor pivotal in regulating insulin release, were also impacted 20. Indeed, the infected mice exhibited a significant reduction in GCK levels (Figure S1E), suggesting a connection between hyperglycemia and decreased GCK levels. Additionally, considering that acute pancreatitis tends to elevate TNF-α levels 21, we investigated whether TNF-α levels displayed an analogous upregulation. As anticipated, the infected mice demonstrated a marked surge in TNF-α levels in contrast to mock control mice lacking EV71 infection (Figure S1F). To sum up, our findings indicate that EV71 infection prompts hyperglycemia through the impairment of pancreatic islets and the subsequent reduction in insulin release in the mouse model.

### Hyperglycemia Potentiates Neurovirulence of EV71

**Figure 1:**
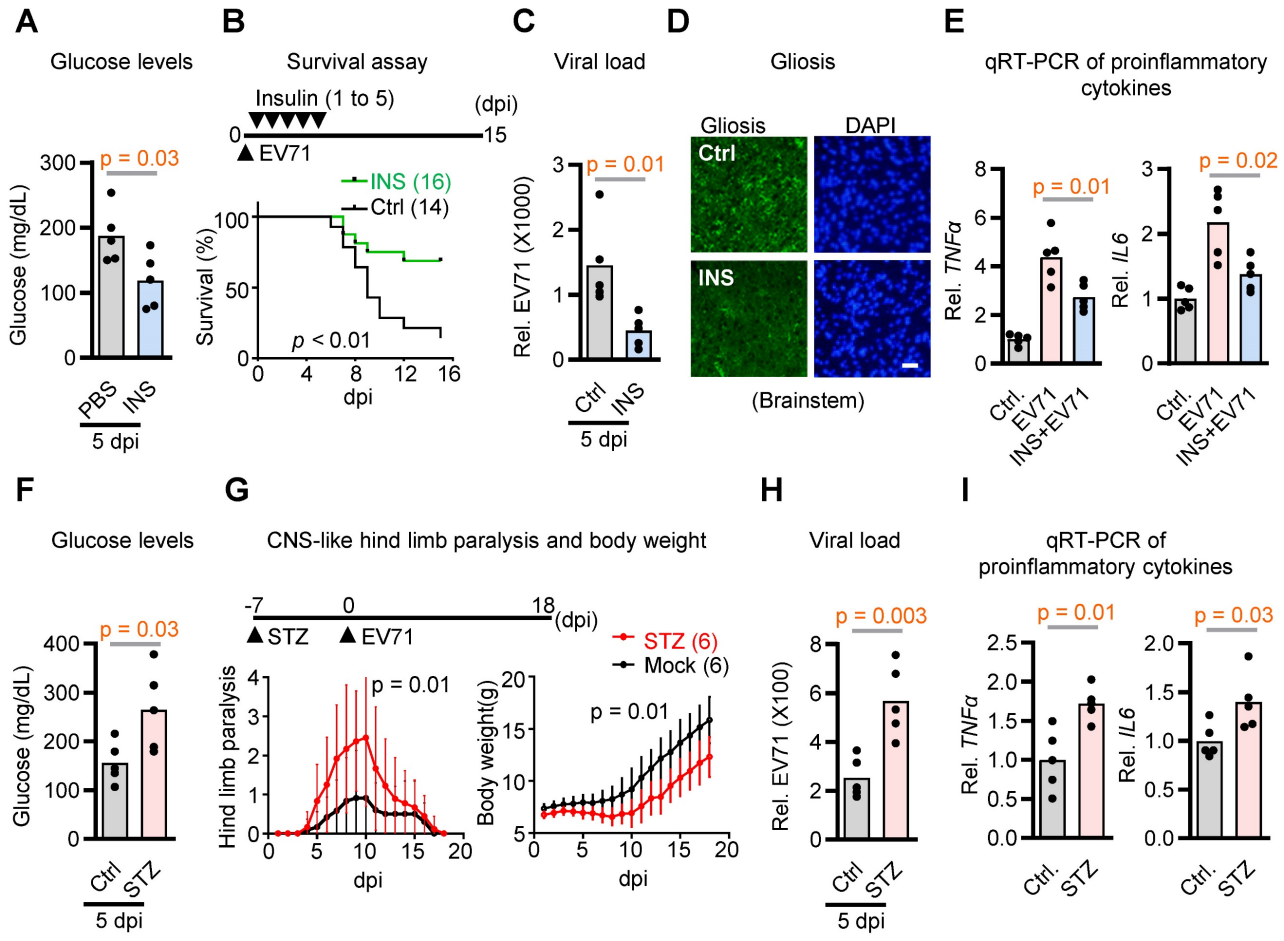
** Contribution of hyperglycemia to neurovirulence in EV71-infected hSCARB2-Tg mice. (A)** Plasma insulin levels in EV71-infected hSCARB2-Tg mice, with or without insulin treatment. Seven-day-old hSCARB2-Tg mice infected with EV71 received subcutaneous injections of long-acting insulin (Ins, 20 ng/kg Insulatard®, n = 5) or PBS (ctrl, n = 5). Mouse Insulin ELISA assay determined plasma insulin levels. Statistical significance was determined using a two-sided T-test, with a significance threshold set at p < 0.05. **(B)** Survival assessment of EV71-infected hSCARB2-Tg mice, with or without insulin treatment. The p-value was calculated using the two-sided log-rank test, with p < 0.05 considered statistically significant. **(C)** Brainstem virus load in EV71-infected hSCARB2-Tg mice, with or without insulin treatment. On day 5 post-infection (dpi), RNA extracted from brainstem tissue was subjected to qRT-PCR analysis using VP1-specific primers and normalized to GAPDH (n = 5). **(D)** Representative micrographs depicting brainstem gliosis in EV71-infected hSCARB2-Tg mice, with or without insulin treatment. Brainstem sections were immunostained with anti-GFAP to evaluate gliosis. Scale bar represents 20 μm. **(E)** Brainstem expression of proinflammatory cytokines in EV71-infected hSCARB2-Tg mice, with or without insulin treatment. RNA extracted from brainstem tissues at 5 dpi was subjected to qRT-PCR analysis using TNFα and IL6-specific primers, normalized to GAPDH. **(F)** Plasma glucose levels in EV71-infected hSCARB2-Tg mice, with or without streptozotocin (STZ) treatment. Fourteen-day-old hSCARB2-Tg mice were injected with STZ (180 mg/kg, n = 5) or PBS (as Ctrl, n = 5), followed by an injection of 3×10^5^ pfu of EV71. Plasma insulin levels determined at 5 dpi. **(G)** CNS-like hind limb paralysis and body weight in STZ- or PBS-treated hSCARB2-Tg mice. The scoring system for CNS diseases ranged from 0 (normal movement) to 5 (severe front and rear limb paralysis). CNS-like hind limb paralysis was defined based on the rigidity of mouse legs. **(H)** Brainstem virus load in EV71-infected hSCARB2-Tg mice, with or without STZ treatment. **(I)** Brainstem expression of proinflammatory cytokines in STZ- or PBS-treated hSCARB2-Tg mice. Values are presented as mean ± SEM.

Glucose availability plays a crucial role in the interplay between viruses and host cells, influencing viral replication and host immune responses 22, 23. However, whether blood glucose acts as an internal constraint for the neurovirulence of EV71 remains uncertain. To test the hypothesis, we administered insulin to EV71-infected mice to correct hyperglycemia and determine its effect on animal survival. The results demonstrated a notable decrease in survival rates among mice during the 0-15 dpi with EV71 in the control group without insulin treatment. Conversely, insulin treatment effectively alleviated hyperglycemia, leading to a substantial improvement in survival rates (Figure 1A-B). Importantly, the viral load in the brainstem was also significantly reduced in response to insulin treatment (Figure 1C). These findings indicate that suppressing hyperglycemia with insulin restrains virus replication in the brainstem and rescues infection-induced lethality.

Building upon our prior investigations that delineated unique neuropathological attributes of EV71 infection in brainstem encephalitis within hSCARB2-Tg mice, encompassing gliosis, escalated proinflammatory cytokine levels, and polio-like paralysis-like symptoms 4, we also evaluated the potential mitigation of these characteristics via insulin treatment. Our findings indeed indicate that the presence of insulin significantly mitigated the levels of gliosis and the proinflammatory cytokines TNFa and IL-6 induced by EV71 infection within the brainstems, in comparison to mock control mice that did not receive insulin treatment (Figure 1D-E). Conversely, when considering streptozotocin-induced hyperglycemia in the SCARB2 transgenic mice, it became evident that this exacerbated various neurological aspects, including hyperglycemia, hind limb paralysis, viral load, and proinflammatory cytokine levels (Figure 1F-I). Collectively, these outcomes suggest that regulating blood glucose levels effectively diminishes the neurovirulence of EV71 in the context of brainstem encephalitis. Thus, it is plausible that hyperglycemia significantly contributes to the heightened neurovirulence of EV71 within the SCARB2 transgenic mouse model.

### Induction of miR-206 Expression in the Brainstem Upon EV71 Infection

**Figure 2:**
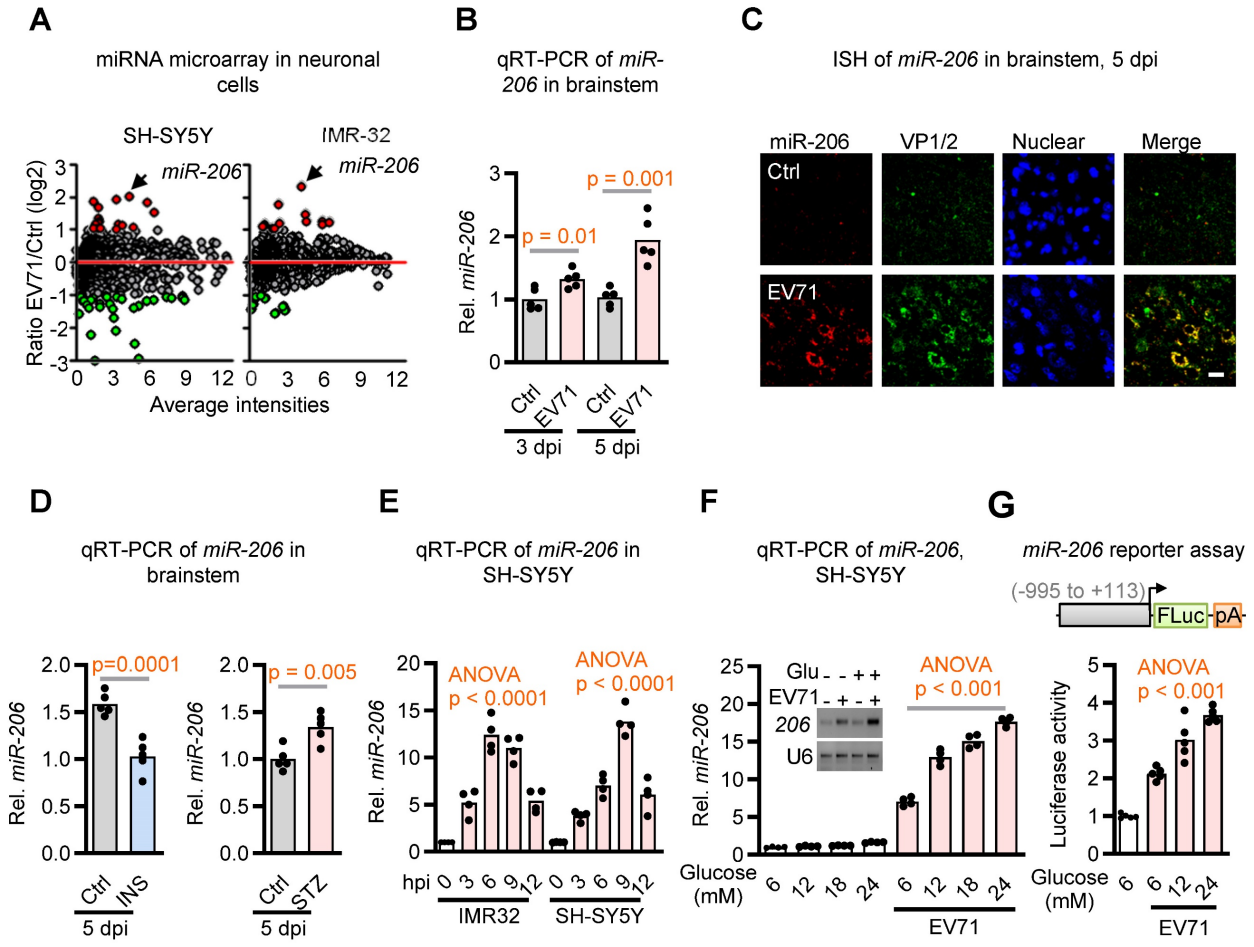
** Identification of miR-206 upregulation in the brainstem during EV71 infection and its modulation by glucose level. (A)** miRNA microarray analysis of two neuroblastoma cell lines infected with EV71. SH-SY5Y and IMR-32 cells were infected with EV71 (MOI = 3) and assessed at 6 h post-infection. Differentially expressed miRNAs were identified through comparative analysis of miRNA expression levels between infected and uninfected cells. **(B)** qRT-PCR analysis of miR-206 expression levels in the brainstem of EV71-infected hSCARB2-Tg mice. Mice (n = 5) were infected with EV71 or left uninfected (Ctrl), and qPCR was performed on isolated total RNA at the indicated dpi. Statistical significance was assessed using a two-sided T-test, with a significance threshold set at p < 0.05. **(C)** miR-206 *in situ* hybridization (ISH) in the brainstem of EV71-infected hSCARB2-Tg mice. The miR-206 probe was utilized to visualize miR-206 expression, while VP-1 antibody labeled EV71-infected cells within brainstem tissue. Scale bar represents 5 μm. **(D)** qRT-PCR assessment of miR-206 expression in the brainstem of EV71-infected hSCARB2-Tg mice under insulin or STZ treatment. qRT-PCR analysis employed primers specific to miR-206 and normalized to GAPDH. Values are presented as mean ± SEM. **(E)** qRT-PCR evaluation of miR-206 expression in EV71-infected IMR32 and SH-SY5Y cells and assayed at the indicated hours post-infection (hpi). **(F)** qRT-PCR measurement of miR-206 expression in EV71-infected cells treated with increasing levels of glucose. SH-SY5Y cells were infected with or without EV71 (MOI = 3) and miR-206 levels were measured at 6 hpi in culture media containing 6, 12, 18, or 24 mM of glucose, with mannitol used for adjustments. **(G)** Luciferase reporter activity of the miR-206 promoter in EV71-infected cells treated with increasing glucose levels. Luciferase reporter activity of the miR-206 promoter (-995 to +113) in EV71-infected cells treated with increasing levels of glucose. SH-SY5Y cells were transfected with a luciferase reporter carrying the miR-206 promoter region. The cells were then infected with or without EV71 (MOI = 3) and exposed to media containing escalating glucose concentrations (5, 12, or 24 mM), with mannitol adjustments. The Promega Luciferase Reporter Assay Kit was employed to conduct the luciferase reporter assay, evaluating miR-206 promoter activity at 6 hpi.

Recent evidence has linked virus infection-induced miRNAs to glucose homeostasis during host-virus interactions 24, 25. To explore this potential association in the context of the dysregulated glucose homeostasis induced by EV71, we conducted an analysis of differentially expressed miRNAs in two neuroblastoma cell lines following EV71 infection by utilizing Affymetrix GeneChip® miRNA Array. Notably, among the array of differentially expressed miRNAs, miR-206, a miRNA recognized for its role in regulating insulin signaling 26, stood out as the singular miRNA displaying substantial upregulation in both neuronal cell lines following EV71 infection (Figure 2A and Table S1). This outcome was subsequently verified through qRT-PCR and *in situ* hybridization (ISH) techniques applied to the brainstems of hSCARB2-Tg mice. Specifically, our findings revealed a notable increase in the expression of miR-206 at 3 dpi, and this increase was further amplified at 5 dpi. Of particular significance, this elevation aligned with the presence of the viral capsid protein VP1/2 within the brainstems subsequent to EV71 infection, as illustrated in Figure 2B-C. Taken together, these findings suggest that the induction of miR-206 is linked to the pathogenesis of EV71-induced brainstem complications.

### Hyperglycemia Mediates Upregulation of miR-206

miR-206 has been implicated in regulating insulin signaling in pancreatic islets 27. Therefore, we aimed to investigate whether the upregulation of miR-206 induced by EV71 in the brainstem is influenced by hyperglycemia. Our findings revealed that insulin treatment significantly suppressed the EV71-induced miR-206 expression, while streptozotocin (STZ) -induced hyperglycemia further enhanced miR-206 expression in the brainstem of hSCARB2-Tg mice compared to the mock control mice (Figure 2D). To ascertain whether EV71 plays a direct role in inducing miR-206 upregulation in neuronal cells, we conducted cell-based assays. Notably, we also detected an elevation in miR-206 expression within EV71-infected SH-SY5Y and IMR-32 cell lines (Figure 2E). Moreover, miR-206 levels increased in a dose-dependent manner with the administration of increasing doses of glucose into the cell culture medium (Figure 2F). To further support these findings, we conducted a miR-206 reporter assay and demonstrated that glucose mediates miR-206 expression by directly upregulating its promoter activity in SH-SY5Y cells (Figure 2G).

Additionally, we investigated whether other less virulent enteroviruses would also upregulate miR-206 in neuronal cells. Notably, SH-SY5Y cells infected with the same virus titers of Coxsackievirus A16 (CV-A16), which is a less virulent strain of enterovirus, exhibited significantly lower levels of miR-206 compared to EV71-infected cells (Figure S2). This finding suggests that the levels of miR-206 depend on the degree of neurovirulence exhibited by the virus. In conclusion, our results indicate that EV71-induced miR-206 expression in neuronal cells is mediated through hyperglycemia.

### miR-206 as a Significant Contributor to the Neurovirulence of EV71

**Figure 3:**
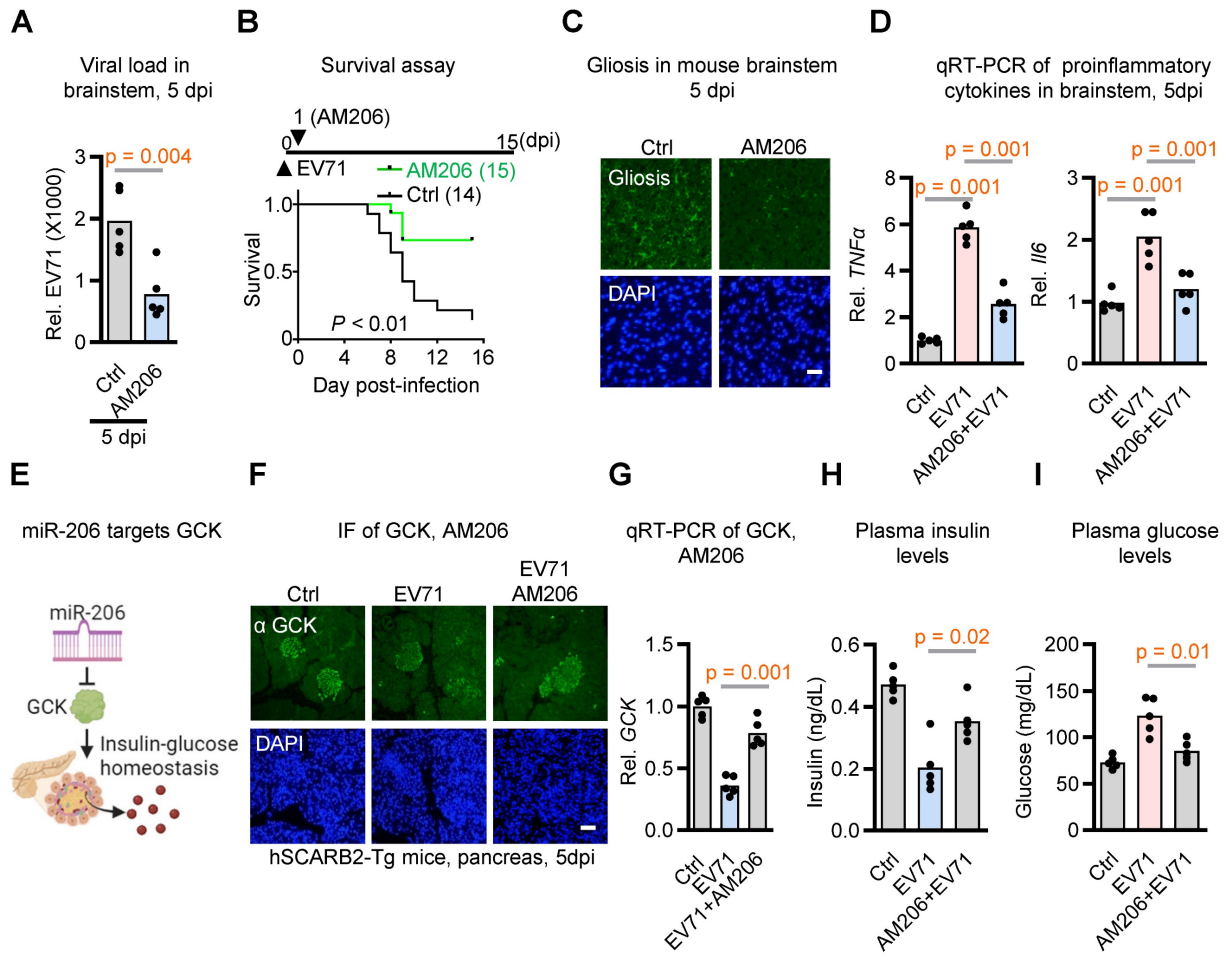
** miR-206 exhibits significant impacts on EV71 neurovirulence and insulin secretion in hSCARB2-Tg mice. (A)** qRT-PCR analysis of viral load in the brainstem of EV71-infected hSCARB2-Tg mice, with or without antagomiR-206 (AM206) treatment (n = 5). Statistical significance was determined using a two-sided T-test, with a significance threshold set at p < 0.05. **(B)** Survival assessment of EV71-infected hSCARB2-Tg mice, with or without AM206 treatment. Seven-day-old hSCARB2-Tg mice infected with EV71 were administered LNA miR-206 antagomir (AM206, 1.2 mg/kg, n = 15) or (Ctrl, n = 14). The log-rank test was utilized to determine the statistical significance in survival rates between the treatment groups. **(C)** Representative micrographs depicting brainstem gliosis in EV71-infected hSCARB2-Tg mice, with or without AM206 treatment. Scale bar represents 20 μm. **(D)** Brainstem expression of proinflammatory cytokines in EV71-infected hSCARB2-Tg mice, with or without AM206 treatment. RNA was extracted from the brainstem tissue of mice at 5 dpi and subjected to qRT-PCR analysis using TNFα-specific, and IL-6-specific primers, normalized to GAPDH (n = 5). **(E)** Proposed role of miR-206 in targeting GCK-mediated insulin-glucose homeostasis. **(F)** Representative immunohistochemistry (IF) images displaying GCK levels in the pancreas of EV71-infected hSCARB2-Tg mice, with or without AM206 treatment. Paraffin-embedded sections were stained using the VP1/2 antibody to detect EV71, while islet staining was performed using an insulin antibody. Scale bar denotes 20 μm. **(G)** qRT-PCR quantification of GCK levels in the pancreas of EV71-infected hSCARB2-Tg mice, with or without AM206 treatment (n = 5). **(H)** Plasma insulin levels in EV71-infected hSCARB2-Tg mice, with or without AM206 treatment. Insulin levels were measured using the mouse Insulin ELISA assay (n = 5). **(I)** Plasma glucose levels in EV71-infected mice, with or without AM206 treatment. Glucose levels were measured using a blood glucose assay (n = 5).

Given that miR-206 is upregulated by EV71-induced hyperglycemia, which enhances virus neurovirulence, we hypothesized that miR-206 signaling might contribute to virus neurovirulence. To test this hypothesis, we administered locked nucleic acid -based antagomiR-206 via i.p. injection to EV71-infected hSCARB2-Tg mice to prevent miR-206 from binding to its target mRNAs. We then examined whether this intervention affected the neurovirulent phenotype. The results supported our hypotheses, as the antagomiR-206 (AM206) treatment led to significant therapeutic effects on neuropathologic changes, including viral load, lethality, gliosis, fatality, and proinflammatory cytokine expressions (Figure 3A-D). These findings suggest that the EV71 infection-induced upregulation of miR-206 in the brainstem plays a key role in promoting the neurovirulent phenotypes of EV71.

### miR-206: A Positive Feedback Loop Intensifying the EV71-induced Hyperglycemia

Considering that miR-206 expression has been demonstrated in pancreatic islets, where it modulates GCK levels in mice exposed to a high-fat diet 26, and given evidence indicating that mutations in the miR-206 binding site reduce its regulatory impact on GCK mRNA, it suggests that GCK could be a direct target of miR-206. Our study aimed to investigate whether elevated miR-206 levels interact to exacerbate EV71-induced hyperglycemia (Figure 3E). To substantiate this hypothesis, we aimed to ascertain if GCK levels were diminished upon EV71 infection and subsequently rescued through the application of the antagomiR-206 AM206. Notably, pancreatic islets within EV71-infected hSCARB2 transgenic mice exhibited a conspicuous decrease in staining signals with the anti-GCK antibody, yet the introduction of antagomiR-206 AM206 significantly reversed the depleted GCK levels (Figure 3F). In support of this observation, our qRT-PCR analysis of GCK also unveiled a decrease in GCK expression levels induced by EV71 infection, which was substantially restored by AM206 within the islets (Figure 3G).

Considering GCK's pivotal role in insulin production and secretion within beta cells, we also assessed plasma insulin and glucose levels. Notably, the reduction in insulin levels triggered by EV71 infection was markedly countered by the administration of AM206 treatment (Figure 3H). Importantly, the utilization of AM206 treatment also effectively mitigated the EV71-induced hyperglycemia (Figure 3I). Collectively, these results indicate that EV71 infection-induced miR-206 establishes a positive feedback loop that exacerbates EV71-induced hyperglycemia within the hSCARB2-Tg mouse model.

### G3BP2 as a Cellular Target of miR-206 in the Brainstem

**Figure 4:**
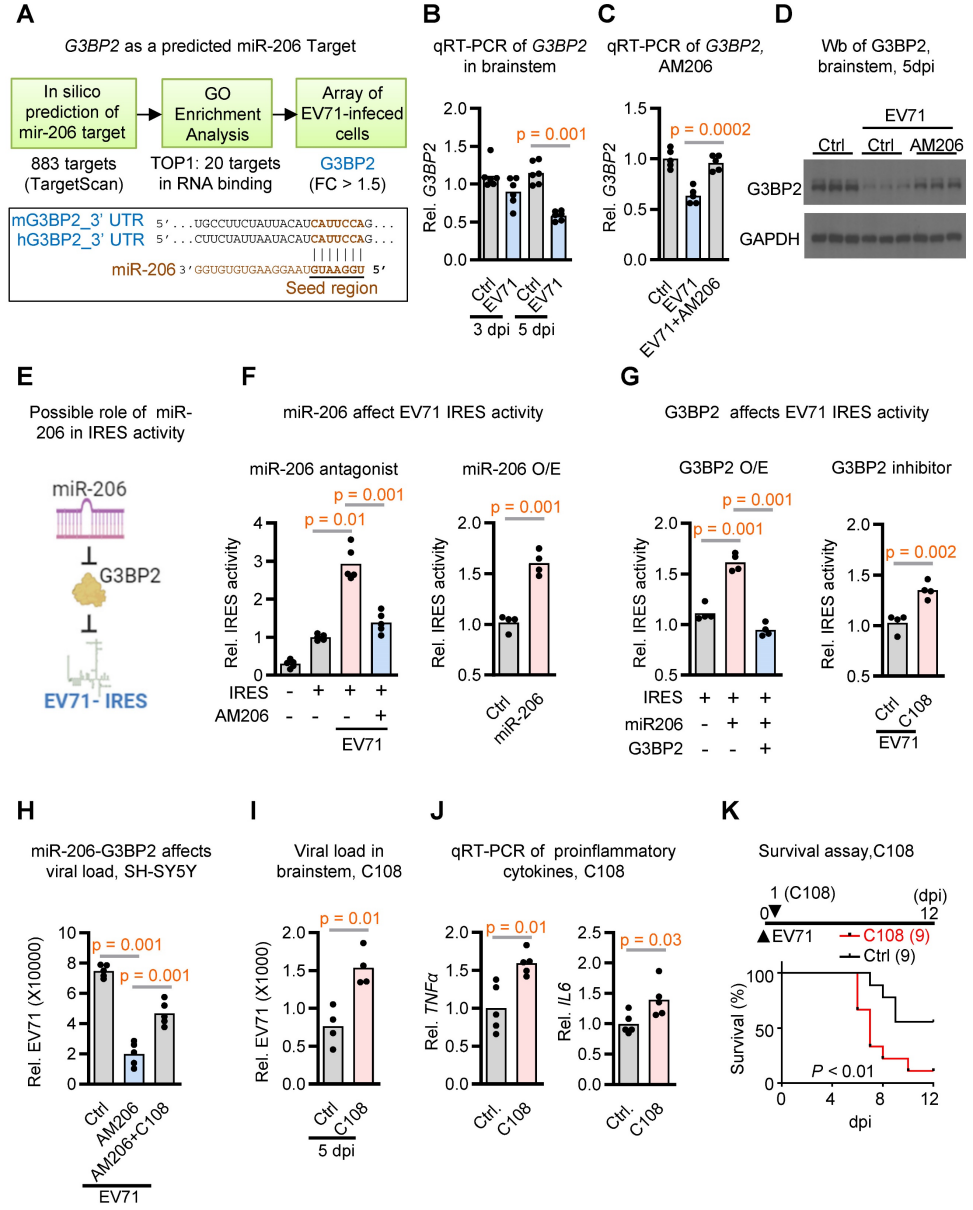
** miR-206 boosting viral replication through G3BP2-mediated activation of virus IRES activity. (A)** Work flow of a threefold approach for identifying potential miR-206 target genes. In silico prediction identified 833 potential candidates using TargetScanVert. GO enrichment analysis identified 20 RNA binding genes among these candidates. Ultimately, microarray analysis of differentially expressed genes in EV71-infected SH-SY5Y cells highlighted G3BP2 as the sole significantly regulated target gene within this RNA-binding gene set. Lower panel depicting sequence alignment between the mature miR-206's seed region and its potential target gene G3BP2, conserved in mouse and human. **(B)** qRT-PCR quantification of G3BP2 expression levels in the brainstem of hSCARB2-Tg with or without EV71 infection. RNA was extracted from the brainstem tissue of mice at 3 or 5 dpi and subjected to qRT-PCR analysis using G3BP2-specific primers, normalized to GAPDH (n = 6). **(C)** qRT-PCR measurement of G3BP2 levels in the brainstem of EV71-infected hSCARB2-Tg mice, with or without AM206 treatment (n = 5). **(D)** Western blot analysis of G3BP2 in the brainstem of EV71-infected hSCARB2-Tg mice, with or without AM206 treatment. Brainstem lysates were subjected to western blotting using G3BP2 and GAPDH antibodies. **(E)** Hypothetical model illustrating miR-206 targeting virus IRES activity through G3BP2. **(F)** Assessment of miR-206's impact on EV71-IRES activity using dual luciferase assay. SH-SY5Y cells were transfected with a luciferase vector containing EV71 IRES, with or without AM206 treatment or miR-206 overexpression. **(G)** Influence of G3BP2 on EV71-IRES Activity. SH-SY5Y cells were transfected with luciferase vector containing EV71 IRES, along with G3BP2 overexpression or the G3BP2 inhibitor C108. **(H)** Dual effects of miR-206 and G3BP3 on virus load. SH-SY5Y cells were treated with miR-206 antagomer AM206, or AM206 in combination with the G3BP2 inhibitor C108, followed by EV71 infection. Virus titers were quantified using qRT-PCR. **(I)** Brainstem viral loads in EV71-infected hSCARB2-Tg mice, with or without C108 treatment. Seven-day-old hSCARB2-Tg mice were subcutaneously injected with the EV71 5746 (C2) strain, followed by C108 (30 mg/kg) injection on day 2, and were subsequent sacrificed on 5 dpi (n = 4). **(J)** qRT-PCR measurement of *TNFα* and *IL6* levels in the brainstem of hSCARB2-Tg mice, with or without C108 treatment (n = 5). **(K)** Survival analysis of EV71-infected hSCARB2-Tg mice, with or without C108 treatment. Seven-day-old hSCARB2-Tg mice infected with EV71 on day 0 were administered C108 (n = 9) or control treatment (n = 9) on day 1. Statistical significance was assessed using the two-sided log-rank test, with p < 0.05 indicating statistical significance.

To unravel the underlying mechanism through which miR-206 contributes to neurovirulence, we employed a comprehensive threefold approach: (1) Initially, by utilizing TargetScan, we identified 883 potential target genes featuring conserved sites that match the seed region of miR-206; (2) From this initial pool of 883 candidates, we pinpointed 20 genes exhibiting the most significant Gene Ontology molecular functions associated with RNA binding; (3) To further refine our focus, we conducted microarray analysis on neuronal cells infected with EV71, revealing that among these 20 RNA binding genes, G3BP2 displayed the sole significant differential regulation (Figure 4A and Table S2).

To substantiate G3BP2's role as a prospective cellular target of miR-206 in the brainstem during EV71 infection, we proceeded with qRT-PCR analysis. Our results showed a decrease in G3BP2 expression levels within brainstem tissues upon EV71 infection (Figure 4B). Importantly, when we impeded miR-206 activity through the use of AM206, we observed the restoration of G3BP2 expression levels (Figure 4C). Furthermore, Western blot analysis revealed a reduction in G3BP2 protein levels following EV71 infection, which was effectively countered by AM206 treatment (Figure 4D). Taken together, these findings provide compelling evidence that G3BP2 might serve as a direct target of miR-206 within the brainstem of EV71-infected mice.

### Initiation of IRES-Driven Viral Replication by miR-206-G3BP2 Signaling

G3BP2 is known to play a crucial role in the regulation of translation initiation, especially through its interactions with IRES elements, which regulate translation under stress conditions 28, 29. Interestingly, the hijacking of G3BP members for IRES-dependent translation appears to be a common phenomenon observed in many RNA viruses, including enterovirus, influenza, dengue, West Nile virus, and Coronavirus disease 2019 30-35. Expanding on these findings, we have developed a hypothesis proposing that miR-206 may influence viral IRES activity via G3BP2 (Figure 4E). To explore this further, we conducted an analysis of EV71 IRES activity in SH-SY5Y cells.

In this experimental setup, cells were transfected with a luciferase vector containing EV71 IRES and subjected to either miR-206 antagomir treatment or miR-206 overexpression. The outcomes from relative IRES activity assessments revealed that miR-206 antagomir AM206 notably reduced EV71 IRES activity initiated by EV71 infection (Figure 4F, left panel). Conversely, miR-206 overexpression heightened EV71 IRES activity (Figure 4F, right panel). These findings support the pivotal role of miR-206 in governing EV71 IRES activity within neuronal cells.

To probe the role of G3BP2 in miR-206-mediated EV71-IRES activity, we conducted EV71 IRES activity assays in cells with or without G3BP2 overexpression, alongside treatment with the G3BP2 inhibitor C108. The results unequivocally demonstrated that while miR-206 overexpression further heightened EV71 IRES activities, this effect was counteracted by G3BP2 overexpression (Figure 4G, left panel). Conversely, treatment with the G3BP2 inhibitor C108 enhanced the EV71 IRES activities induced by miR-206 overexpression (Figure 4G, right panel). Additionally, we sought to determine whether the reduction in viral loads caused by miR-206 antagomir treatment could be counteracted by the G3BP2 inhibitor. Remarkably, the G3BP2 inhibitor effectively reversed the impact of miR-206 antagomir (Figure 4H). Collectively, these cell-based assays provide compelling support for the concept that the miR-206-G3BP2 signaling axis plays a pivotal role in modulating EV71 IRES activity within neuronal cells.

Moving beyond cell-based assays, we extended our validation to infected mice by administering the G3BP2 inhibitor and assessing its effects on viral load and animal survival. Strikingly, the treatment led to a significant increase in virus loads and a higher rate of infection-induced animal mortality (Figure 4I-K). Taken together, these findings underscore the crucial role of elevated miR-206, induced by EV71 infection-triggered hyperglycemia, in enhancing EV71 IRES activity. This enhancement is achieved through the suppression of its target gene G3BP2, which in turn plays a significant role in the intricacies of stress granule formation.

## Discussion

The major impact of this study on the infectious disease field lies in the understanding that virus-host interaction-induced metabolic changes are pivotal in enhancing virus neurovirulence. Notably, the CNS presents an inhospitable setting for virus infiltration and replication, characterized by a restricted immune response and diminished potential for cellular recovery and rejuvenation. As a result, when a virus invades the CNS, it faces a distinctive microenvironment with specific challenges distinct from other body tissues, affecting its ability for efficient viral replication. To navigate the host's metabolic limitations, RNA viruses such as enteroviruses must increase host glucose levels to facilitate efficient viral protein synthesis. Take neurovirulent EV71 as an example; this study illustrates the reduction of hyperglycemia using insulin alone was observed to significantly lower viral load and mitigate the pathological manifestations of encephalitis in the EV71-infected mice. This highlights the crucial role of hyperglycemia as a fundamental factor in the neurovirulence of EV71 infections, underscoring the potential application of regulating blood glucose levels to enhance outcomes for individuals with encephalitis.

However, simply increasing the host's glucose supply is not enough for efficient virus replication. In fact, hyperglycemia not only fails to support the regular cellular translation machinery but also paradoxically inhibits the cap-dependent translation machinery, thereby affecting both cellular and viral protein synthesis 36. Therefore, single-stranded RNA enteroviruses must employ an alternative translation mechanism to surpass this translational restriction, enabling them to amplify their replication and spread within the host. Importantly, we provide evidence that virus infection-induced hyperglycemia also triggers an IRES-dependent translation initiation mechanism, facilitating the synthesis of viral proteins independently from the cap-dependent translation initiation pathway.

In terms of mechanism, we have substantiated the significant role of miR-206 in initiating IRES-dependent translation of viral proteins. This suggests that inhibiting miR-206's interaction with its target mRNAs could potentially curtail viral replication and attenuate its neurovirulence. Indeed, the therapeutic potential was demonstrated by administering antagomiR-206 to infected mice, resulting in notable improvements in neuropathological features, including viral load, lethality, gliosis, fatality, and proinflammatory cytokine expression. Further exploration of miR-206's mechanistic role in inducing IRES activity unveiled G3BP2 as a pivotal cellular target modulated by miR-206. This regulatory influence extends to both EV71 IRES activity and viral neurovirulence. Given that the suppression of G3BP stress granule proteins is also essential for IRES-mediated virus replication in numerous other RNA viruses 37, 38, it would be intriguing in the future to investigate whether the miR-206-stress granule signaling pathway is similarly conserved across other RNA viruses.

While G3BP2 indeed serves as a crucial cellular target for miR-206-mediated IRES activity during EV71 infection, it is noteworthy that other cellular target mRNAs might also be influenced by miR-206. A single miRNA can effectively target multiple genes, orchestrating various cellular processes in response to environmental stimuli, thereby finely tuning their expression levels and overall response 39. Consequently, it is plausible that hyperglycemia-induced miR-206 might simultaneously target additional cellular genes to modulate IRES activity. Considering the replication of enteroviruses within autophagosomes, it's reasonable to speculate that IRES-dependent viral replication occurs within these structures. As such, cellular factors impacting autophagosomes could also be subject to miR-206's influence. This speculation is particularly intriguing given that miR-206 actively modulates insulin signaling, which is known to negatively regulate autophagy. Hence, future investigations could explore whether miR-206 also targets cellular factors involved in autophagosome formation, potentially promoting IRES-dependent virus replication within autophagosomes.

Furthermore, besides its impact on miR-206-mediated promotion of efficient virus proliferation within host cells, hyperglycemia acts as a robust inducer of cellular metabolic pathways, notably glycolysis 40. In response to heightened glucose availability, virus-infected cells upregulate glycolytic enzymes and transporters, facilitating increased glucose uptake and metabolism 41. Consequently, glucose undergoes rapid glycolytic metabolism, yielding pyruvate and ATP. This enhanced ATP production not only fuels viral replication processes but also meets the heightened metabolic demands associated with viral infection 42. Additionally, intermediates generated during glycolysis serve as precursors for the biosynthesis of macromolecules essential for viral replication, including nucleotides, amino acids, and lipids. Thus, the initiation of the glycolytic pathway following hyperglycemia induction in virus-infected cells plays a pivotal role in promoting efficient viral replication and propagation within the host cellular environment. Interestingly, miR-206 has been reported to modulate glycolysis in cancer cells 43, 44, suggesting potential interplay between hyperglycemia-induced metabolic alterations and miR-206-mediated effects, further influencing viral proliferation. However, we observed an unexpected downregulation of three Glut genes (Glut1, Glut4, and Glut7) in EV71-infected cells, regardless of glucose concentration (Figure S3). One possibility is that decreased Glut expression could induce endoplasmic reticulum (ER) stress, potentially enhancing viral protein production and replication 45, 46. Further investigation is needed to elucidate the interplay between hyperglycemia, Glut regulation, and EV71 infection.

Focusing on cellular mechanisms exploited by viruses presents distinct advantages in treating emerging viruses, as opposed to directly targeting viral proteins. Cellular targets exhibit greater resistance to mutational changes over time, ensuring stability and suitability for development of therapeutics 47. This stability reduces the chances of mutational escape and minimizes the risk of drug resistance, especially during the emergence of new viruses 48. An added benefit of targeting the hyperglycemia-miR-206 signaling pathway lies in its potential applicability to other enterovirus strains like D68 and polio. This implies that inhibiting the hyperglycemia-miR-206 signaling-mediated IRES activity could serve as a comprehensive intervention strategy for virus-induced encephalitis.

## Methods

### hSCARBT2- mice

At 7 or 14 days of age, juvenile hSCARB2-Tg (transgenic) and non-TG wild-type mice were subjected to subcutaneous inoculation with EV71 5746 strains or medium alone. Mice were allocated randomly in all experiments. For Insulin treatment study, seven-day-old hSCARB2-Tg mice infected with 3X10^4^ pfu of EV71 received subcutaneous injections of long-acting insulin (Ins, 20 ng/kg Insulatard®) or PBS (ctrl) once daily for five days. For the Streptozocin (STZ) treatment study, s Seven-day-old hSCARB2-Tg mice received intraperitoneal (IP) treatment with STZ (180 mg/kg, Sigma) once daily for five days. At 14 days of age, mice were infected with 3X10^5^ pfu of EV71. For C108 treatment study, Seven-day-old hSCARB2-Tg mice infected with 3X10^4^ pfu of EV71and received IP injections of C108 (30 mg/kg, Cayman Chemical) every other day. For the miR-206 antagomir study, seven-day-old hSCARB2-Tg mice infected with 3X10^4^ pfu of EV71 received intraperitoneal (IP) injections of miR-206 antagomir (AM206, CsCsACACACACUUCCUUACAUUs CsC sAs-Chol, TAQKEY Science) at a dosage of 1.2 mg/kg, while a control group was administered with control antagomir (Ctrl, CsAsCGGUUCCAGGCACUGUsG sU s As -Chol-3). Following the treatment period, comprehensive analyses were conducted on all animals, encompassing the monitoring of expression of viral VP1, mouse body weight, assessment of central nervous system (CNS) syndromes, and determination of mouse survival rates. Severity of CNS syndromes was graded on a scale of 0 to 5, utilizing the following criteria: 5  =  severe front and rear limb paralysis (LP) with no movement, 4  =  moderate with 2 rear LP and hesitant movement, 3  =  one rear LP with bending legs, 2  =  mild rear limb bending, 1  =  slightly rear limb bending, 0  =  normal movement. LP is defined as the rigidity of mouse legs that exhibit hesitation in movement. The log-rank test was utilized to evaluate the statistical significance in survival rates between the treatment and control groups. All procedures and protocols involving experimental animals were approved by the Institutional Animal Care and Use Committee at NHRI (approved protocol no. NHRI-IACUC-099007-A).

### Measurement of Plasma Insulin and Glucose Levels

In EV71-infected mice, plasma glucose levels were quantified using a blood glucose assay with the FUJIFILM DRI-CHEM NX500 system. Insulin levels were determined using the Mouse INS (Insulin) ELISA Kit by Elabscience.

### Immunostaining Assay and Western Blot Immunoassays

Immunostaining assays were performed following previous protocols 4. Briefly, brainstem tissues were collected, fixed in 4% paraformaldehyde for 12 h, and then embedded in paraffin wax. Paraffin sections were deparaffinized, rehydrated, and subjected to antigen retrieval. Subsequently, the sections were treated with a blocking reagent and incubated overnight at 4°C with specific primary antibodies. On the following day, the sections were exposed to a fluorescence-conjugated secondary antibody for 1 h at RT and visualized using a fluorescent microscope.

### Cell culture and transfection

The following cell lines were used in this study: SH-SY5Y (human neuroblastoma, ATCC CRL-2266), IMR-32 (human neuroblastoma, ATCC CCL-127). SH-SY5Y and IMR32 were cultured in MEM (Invitrogen, USA). Cells were grown at 37°C in a 5% CO2 humid atmosphere. RNAi‐mediated knockdown of miR-206 was performed by transient transfection of miR-206 inhibitor (CACACACTTCCTTACATTCC, EXIQON) or a control siRNA into cells with DharmaFECT (Dharmacon) at a concentration of 50 nM in 6‐well culture plates. Transient overexpression of G3BP2 in cells was performed by transfecting a G3BP2 expression plasmid (pCDNA3-G3BP2) or a mock control plasmid (pCDNA3) with Lipofectamine 2000 (Invitrogen).

### In Situ Hybridization Assay of miR-206

Fluorescence in situ hybridization was used to identify the localization and expression pattern of miR-206 in the tissues of EV71-infected hSCARB2-Tg mice, following a previously described method 38. LNA-modified microRNA miR-206 antisense oligonucleotide probes were labeled with Biotin (MDBio Inc.) and the signal was amplified using Tyramide amplification solution (Thermo Fisher Scientific). The tissue sections on the slides were first incubated in an oven at 47 °C overnight for optimal sample conditions. Sections then underwent dewaxing in xylene (5 min, 3 times) and subsequent rehydration through an ethanol series (100% 10 min, 2 times; 95% 10 min; 70% 5 min; 50% 5 min; 30% 5 min; dH2O 5 min, 2 times). The slides were then treated in 10 mM sodium citrate buffer (pH 6.0) at room temperature (RT), brought to a boil for 10 min, followed by cooling for 30 min. Washing steps involved dH2O (5 min, 3 times with gentle shaking) and PBS (5 min with gentle shaking) to ensure effective removal of residual elements. Prehybridization treatment included the use of a liquid-repellent marker and blocking in prehybridization buffer (0.03 g BSA, 200 μl 20× SSC and 800 μl water) for 20 min at RT. Subsequently, hybridization with the miR-206-specific probe occurred in hybridization buffer (0.25 g dextran sulfate sodium salt, 0.5 ml 20× SSC, 2 ml water and LNA oligonucleotide probes at 1-2 ng/ml) for 1 h at room temperature. Washing included Washing buffer I (30 °C, 5 min, 3 times, 4× SSC, 0.1% Tween-20) and Washing buffer II (30 °C, 5 min, 2× SSC). Signal amplification involved incubation with hydrogen peroxide solution ((20 ml 30% H2O2 and 180 ml PBS)) for 20 min at RT, washing with TN buffer ((0.1 M Tris-HCl pH 7.5, 0.15 M NaCl)), and TNB blocking buffer (0.05 g blocking reagent (BSA) in 10 ml TN buffer) treatment 30 min at RT. Detection included incubation with Streptavidin/HRP solution (1 μl Streptavidin/HRP in 500 μl TNB buffer) for 30 min at RT, washing with TNT buffer (0.1-M Tris-HCl pH 7.5, 0.15-M NaCl, 0.2% (vol/vol) Triton X-100), and incubation with tyramide amplification solution (8 μl Biotin stock solution in 400 μl plus Amplification Diluent) for 10 min at RT. Primary antibody solution preparation (200 μl) and incubation overnight at 4 °C (0.02 g BSA, 2 ul VP1 antibody + 2 ul SA-HRP in 1ml PBS ). Subsequent washing with 1× PBS and secondary antibody solution incubation (0.02 g BSA, 10 ul Anti-mouse FITC, 10 ul HRP-TRITC, 10 ul Anti-rabbit Cy5 and 2 ul DAPI in 1ml PBS) for 45-60 min at RT. Covering with an appropriate mounting medium, drying by standing slides upright, waiting for at least 1 h before viewing, storing overnight at 4 °C in the dark, and sealing coverslips with nail polish ensured the completion of the assay. Control experiments were carried out using a scrambled miRNA probe to validate assay specificity. Visualization of miR-206 and VP1 expression was conducted using a fluorescent microscope, and images were captured for further analysis and characterization of miR-206 distribution and abundance in the investigated tissues or cells.

### Quantitative Real-Time Reverse Transcription-PCR

Total RNAs were extracted from brainstem tissues or cultured cells using the illustra™ RNAspin Mini RNA Isolation Kit (GE Healthcare Life Sciences) following the manufacturer's instructions. A starting amount of 1 μg total RNA was used for subsequent cDNA synthesis with the High-Capacity cDNA Reverse Transcription Kits (ABI Applied Biosystems, USA). For quantitative real-time reverse transcription-PCR (qRT-PCR) analysis, the Fast SYBR Green Master Mix (ABI Applied Biosystems, USA) was employed. Gene expression levels were determined utilizing the relative standard curve method, providing a quantitative measure of mRNA expression in the samples. The primer used in this study: TNFα: 5ʹ-CTACTCCCAGGTTCTCTTCAA-3ʹ and 5ʹ-GCAGAGAGGAGGTTGACTTTC-3ʹ; IL6: 5ʹ-TACCACTTCACAAGTCGGAGGC-3ʹ and 5ʹ-CTGCAAGTGCATCATCGTTGTTC-3ʹ; pan-enterovirus-specific primer: 5ʹ-GTGTGAAGAGTCTATTGAGC-3ʹ and 5ʹ-ATTGTCACCATAAGCAGCCA-3ʹ; GAPDH: 5ʹ-CATCACTGCCACCCAGAAGACTG-3ʹ and 5ʹ-ATGCCAGTGAGCTTCCCGTTCAG-3ʹ; 5'-GCK: GCATCTCTGACTTCCTGGACAAG-3' and 5'-CTTGGTCCAGTTGAGCAGGATG-3'; G3BP2: 5'-GCATCTCTGACTTCCTGGACAAG-3' and 5'-CTTGGTCCAGTTGAGCAGGATG-3'.

miR-206 levels were measured using the TaqMan miR-206 miRNA Assay kit (ABI, Cat# 4427975), following the manufacturer's instructions. A starting amount of 0.5 μg total RNA was used for subsequent cDNA synthesis with the Superscript III Reverse Transcriptase kit (Thermo Fisher Scientific). Real-time PCR was performed using a standard TaqMan® PCR kit protocol on an Applied Biosystems StepOnePlus System (Applied Biosystems). The 10 µl PCR included 0.2 µl RT product, 1× TaqMan® Universal PCR Master Mix (Applied Biosystems), 0.2 µM TaqMan® probe, 1.5 µM forward primer and 0.7 µM reverse primer. The reactions were incubated in a 96-well plate at 95°C for 10 min, followed by 40 cycles of 95°C for 15 s and 60°C for 1 min. All reactions were run in triplicate. The threshold cycle (CT) is defined as the fractional cycle number at which the fluorescence passes the fixed threshold. TaqMan® CT values were converted into absolute copy numbers using a standard curve from synthetic miR-206 miRNA.

### miRNA Microarray

To identify key miRNAs differential expressed in neuronal cells infected by EV71, miRNA microarray experiments were conducted using two distinct cell lines, namely SH-SY5Y and IMR-32 neuroblastoma cells. Total RNA, encompassing miRNAs, was isolated through a combination of TRIzol and the RNeasy mini kit (Qiagen). In detail, the cells or tissue were homogenized in 800 μl TRIzol, followed by the addition of 200 μl chloroform. After incubation and centrifugation, the upper phase was extracted and mixed with 0.53 times the volume of 100% ethanol. This mixture was then applied to the miRNeasy mini kit column, following the manufacturer's protocol. For miRNA microarray analysis, the Affymetrix GeneChip miRNA array was employed in the NHRI Microarray Core facility, adhering to the manufacturer's instructions. The chip quality control report generated by the Expression Console Software of Affymetrix Company included assessments of quality control results, detection of spikes in the probe, and evaluation of signal values. Subsequently, data underwent normalization, and differential expression analysis of miRNAs was performed.

### Quantification and Statistical Analysis

The sample sizes (number of patients or mice), means, and statistical significance values can be found in the Figure legends. To ensure the validity and reliability of the results, all experiments were carried out in triplicate or more. Statistical comparisons assessing the significance of observed differences among all groups were performed using GraphPad Prism 10.

## Supplementary Material

Supplementary figures and tables.

## Abbreviations

AM206: antagomiR-206
CV-A16: Coxsackievirus A16
CNS: central nervous system
COVID-19: Coronavirus disease 2019
EV71: Enterovirus 71
G3BP2: G3BP stress granule assembly factor 2
GCK: Glucokinase
hSCARB2: human scavenger receptor class B member 2
IL-6: Interleukin 6
IRES: internal ribosome entry site
ISH: in situ hybridization
LNA: Locked Nucleic Acid
qRT-PCR: Quantitative reverse transcription polymerase chain reaction
RNA: Ribonucleic acid
STZ: Streptozotocin
Tg: transgenic
TNFa: Tumor necrosis factor alpha
UTR: untranslated regions
VP1/2: viral protein 1/2
