# Supplementary material for: Hyperglycemia facilitates EV71 replication: Insights into miR-206-mediated regulation of G3BP2 promoting EV71 IRES activity
Source: Theranostics. 2024 Apr 22;14(7):2706–18. doi: 10.7150/thno.93883 (PMC11103504; doi:10.7150/thno.93883)
Supplement: Supplementary file 1 — Supplementary figures and tables. [file thnov14p2706s1.pdf]

Supplementary Materials

Figure. S1.

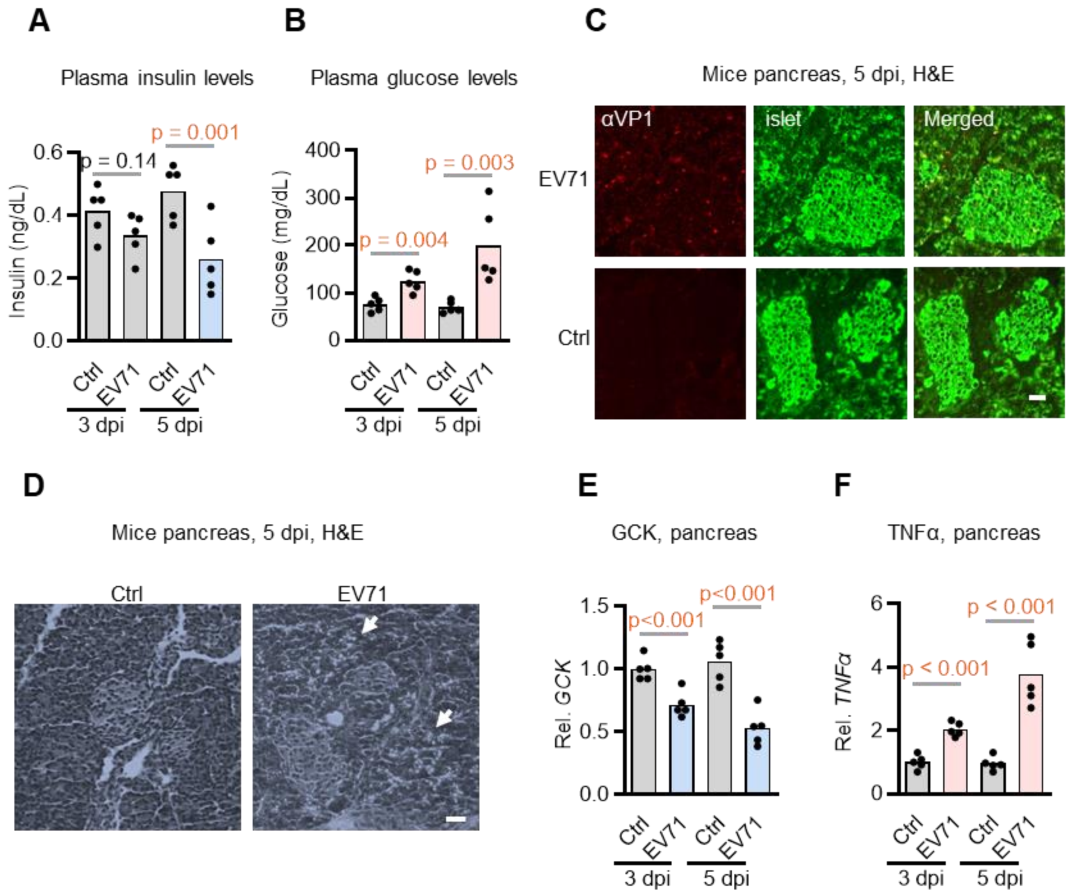

Figure S1. EV71 Infection Disrupts Insulin Release in hSCARB2-Tg Mice

(A) (B) Plasma insulin and glucose levels in EV71-infected mice (n = 5). Plasma glucose levels were measured at 3 and 5 dpi using a blood glucose assay (a), and insulin levels were determined using the mouse Insulin ELISA assay (b). Statistical significance was evaluated using a two-sided T-test, with a significance threshold of $p < 0.05$. **(C)** Representative immunohistochemistry images illustrating the distribution of EV71 VP1 proteins in the pancreas of hSCARB2-TG mice with or without EV71 infection. Paraffin-embedded sections were stained using the VP1/2 antibody to visualize EV71, while islet staining was conducted with an insulin antibody. Scale bar indicates 20 µm. **(D)** Histopathological hematoxylin and eosin (H&E) examination highlighting EV71-induced lesions in the pancreas of hSCARB2-Tg mice with or without EV71 infection. Scale bar indicates 20 µm. **(E) (F)** qRT-PCR analysis of GCK and TNFα expression levels in the pancreas of EV71-infected mice. Seven-day-old hSCARB2-Tg mice (n = 5) were subcutaneously administered 3×10^4 pfu of the EV71 5746 (C2) strain, followed by sacrifice at indicated time points. Specific primers for GCK and TNFα were employed for qRT-PCR analysis, with GAPDH serving as the normalization control.

Figure. S2.

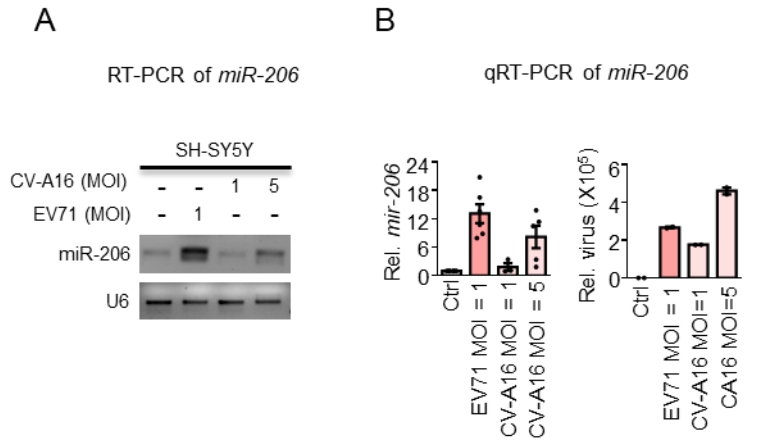

Figure S2. Attenuated Induction of miR-206 Expression by Coxsackievirus A16

(A) Agarose gel analysis of RT-PCR products of miR-206. SH-SY5Y cells were infected with EV71 (MOI = 1), or CV-A16 (MOI = 1 or 5), or left uninfected.

(B) qRT-PCR analysis of miR-206 expression levels and virus titers using primers specific to miR-206 and virus VP1. Data normalized to GAPDH are represented as the mean ± SEM.

Figure. S3.

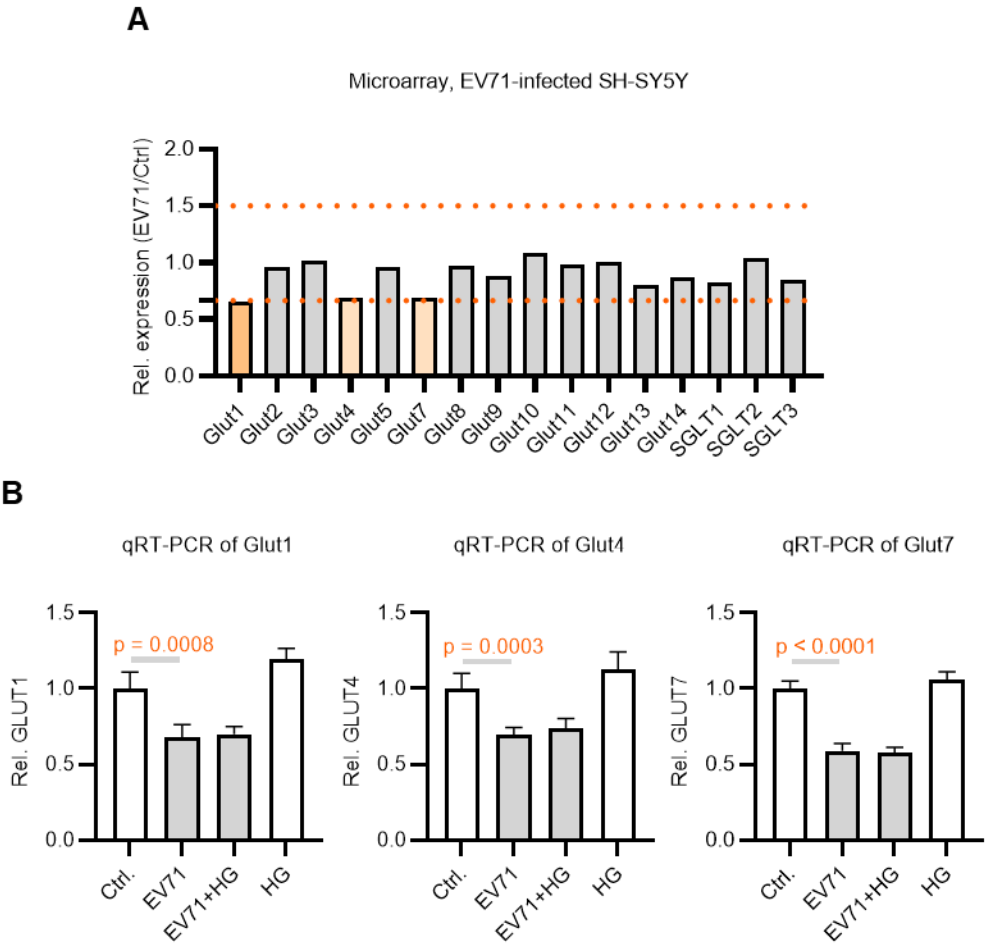

Figure S3. Glucose transporters (Gluts) levels in EV71-infected neuronal-like cell lines. (A) Microarray analysis of glucose transporter genes in EV71-infected SHS-Y5Y cells compared to Mock cells. (B) qRT-PCR of Glut1, Glut4 and Glut7 in EV71-infected SHS-Y5Ycells with or with high glucose treatment.

Table S1. miRNA Microarray Analysis of EV71-Infected neuroblastoma cells.

Fold change (FC) is determined by normalization against uninfected cells.

SH-SY5Y (51)						IMR32 (22)			
**miRNA**	**FC#**	**miRNA**	**FC**	**miRNA**	**FC**	**miRNA**	**FC**	**miRNA**	**FC**
**miR-206**	**4.08**	miR-941	-7.98	miR-744	-2.25	**miR-206**	**5.00**	miR-1306	-2.99
miR-510	3.78	miR-210	-7.74	miR-200c	-2.24	miR-1238	3.40	miR-377	-2.82
miR-186	3.61	miR-331-5p	-5.63	miR-324-5p	-2.23	miR-885-3p	2.77	miR-1322	-2.76
miR-1246	3.60	miR-21	-4.68	miR-32	-2.22	miR-767-3p	2.39	miR-1263	-2.55
miR-222	3.21	miR-887	-4.25	miR-490-5p	-2.22	miR-361-3p	2.38	miR-890	-2.46
miR-1281	2.90	miR-720	-3.23	miR-675	-2.16	miR-210	2.33	miR-615-5p	-2.17
miR-886-3p	2.53	miR-526a	-3.00	miR-423-3p	-2.14	miR-34c-5p	2.29	miR-647	-2.15
miR-521	2.39	miR-1303	-2.69	miR-125a-3p	-2.13	miR-15b	2.26	miR-601	-2.08
miR-300	2.28	miR-101	-2.65	miR-590-5p	-2.13	miR-548c-3p	2.22	miR-548b-3p	-2.06
miR-483-5p	2.25	miR-22	-2.61	miR-665	-2.09	miR-512-5p	2.17		
miR-376a	2.24	miR-187	-2.59	miR-506	-2.09	miR-499-5p	2.13		
miR-374b	2.22	miR-550	-2.50	miR-140-3p	-2.07	miR-148a	2.07		
miR-572	2.09	miR-95	-2.48	miR-487a	-2.07	miR-664	2.05		
miR-299-5p	2.05	miR-29c	-2.44	miR-342-5p	-2.06				
miR-384	2.05	miR-921	-2.42	miR-485-3p	-2.06				
miR-520h	2.05	miR-33a	-2.26	miR-518f	-2.03				
miR-326	2.02	miR-548l	-2.26	miR-1270	-2.00				

Table S2. GO molecular function of potential targets of miR-206.

GO molecular function	Total	miR-206 targets	Fold Enrichment	raw P-value	FDR
**RNA (mRNA) binding (GO:0003729)**	**142**	**20**	**3.29**	**1.12E-05**	**3.08E-04**
actin binding (GO:0003779)	139	19	3.2	2.69E-05	5.16E-04
sequence-specific DNA binding RNA polymerase II transcription factor activity (GO:0000981)	231	26	2.63	2.25E-05	5.41E-04
chromatin binding (GO:0003682)	177	19	2.51	7.33E-04	1.08E-02
kinase activity (GO:0016301)	625	53	1.98	7.31E-06	2.34E-04
protein kinase activity (GO:0004672)	414	33	1.86	1.06E-03	1.46E-02
sequence-specific DNA binding transcription factor activity (GO:0003700)	805	62	1.8	2.28E-05	4.86E-04
DNA binding (GO:0003677)	1084	82	1.77	2.03E-06	9.75E-05
nucleic acid binding (GO:0003676)	1625	114	1.64	5.39E-07	3.45E-05
transferase activity (GO:0016740)	1325	87	1.54	1.30E-04	2.26E-03
